# CAGI6 ID-Challenge: Assessment of phenotype and variant predictions in 415 children with Neurodevelopmental Disorders (NDDs)

**DOI:** 10.21203/rs.3.rs-3209168/v1

**Published:** 2023-08-02

**Authors:** Maria Cristina Aspromonte, Alessio Del Conte, Shaowen Zhu, Wuwei Tan, Yang Shen, Yexian Zhang, Qi Li, Maggie Haitian Wang, Giulia Babbi, Samuele Bovo, Pier Luigi Martelli, Rita Casadio, Azza Althagafi, Sumyyah Toonsi, Maxat Kulmanov, Robert Hoehndorf, Panagiotis Katsonis, Amanda Williams, Olivier Lichtarge, Su Xian, Wesley Surento, Vikas Pejaver, Sean D. Mooney, Uma Sunderam, Rajgopal Srinivasan, Alessandra Murgia, Damiano Piovesan, Silvio C. E. Tosatto, Emanuela Leonardi

**Affiliations:** Department of Biomedical Sciences, University of Padova; Department of Biomedical Sciences, University of Padova; Department of Electrical and Computer Engineering, Texas A&M University, College Station, TX 77843; Department of Electrical and Computer Engineering, Texas A&M University, College Station, TX 77843; Department of Electrical and Computer Engineering, Texas A&M University, College Station, TX 77843; CUHK Shenzhen Research Institute, Shenzhen; CUHK Shenzhen Research Institute, Shenzhen; CUHK Shenzhen Research Institute, Shenzhen; Biocomputing Group, Department of Pharmacy and Biotechnology, University of Bologna; Department of Agricultural and Food Sciences, University of Bologna; Biocomputing Group, Department of Pharmacy and Biotechnology, University of Bologna; Biocomputing Group, Department of Pharmacy and Biotechnology, University of Bologna; Computational Bioscience Research Center (CBRC), Computer, Electrical and Mathematical Sciences & Engineering Division (CEMSE), King Abdullah University of Science and Technology (KAUST), Thuwal 23; Computational Bioscience Research Center (CBRC), Computer, Electrical and Mathematical Sciences & Engineering Division (CEMSE), King Abdullah University of Science and Technology (KAUST), Thuwal 23; Computational Bioscience Research Center (CBRC), Computer, Electrical and Mathematical Sciences & Engineering Division (CEMSE), King Abdullah University of Science and Technology (KAUST), Thuwal 23; Computational Bioscience Research Center (CBRC), Computer, Electrical and Mathematical Sciences & Engineering Division (CEMSE), King Abdullah University of Science and Technology (KAUST), Thuwal 23; Department of Molecular and Human Genetics, Baylor College of Medicine, One Baylor Plaza, Houston, TX 77030; Department of Molecular and Human Genetics, Baylor College of Medicine, One Baylor Plaza, Houston, TX 77030; Department of Molecular and Human Genetics, Baylor College of Medicine, One Baylor Plaza, Houston, TX 77030; Department of Biomedical Informatics and Medical Education, University of Washington, Seattle, WA 98195; Department of Biomedical Informatics and Medical Education, University of Washington, Seattle, WA 98195; Institute for Genomic Health, Icahn School of Medicine at Mount Sinai, New York, NY 10029; Department of Biomedical Informatics and Medical Education, University of Washington, Seattle, WA 98195; Innovation Labs, Tata Consultancy Services, Hyderabad; Innovation Labs, Tata Consultancy Services, Hyderabad; Department of Women’s and Children’s Health, University of Padova; Department of Biomedical Sciences, University of Padova; Department of Biomedical Sciences, University of Padova; Department of Biomedical Sciences, University of Padova

**Keywords:** CAGI, gene-panel, variants interpretation, phenotype prediction, Neurodevelopmental Disorders

## Abstract

In the context of the Critical Assessment of the Genome Interpretation, 6th edition (CAGI6), the Genetics of Neurodevelopmental Disorders Lab in Padua proposed a new ID-challenge to give the opportunity of developing computational methods for predicting patient’s phenotype and the causal variants. Eight research teams and 30 models had access to the phenotype details and real genetic data, based on the sequences of 74 genes (VCF format) in 415 pediatric patients affected by Neurodevelopmental Disorders (NDDs). NDDs are clinically and genetically heterogeneous conditions, with onset in infant age. In this study we evaluate the ability and accuracy of computational methods to predict comorbid phenotypes based on clinical features described in each patient and causal variants. Finally, we asked to develop a method to find new possible genetic causes for patients without a genetic diagnosis. As already done for the CAGI5, seven clinical features (ID, ASD, ataxia, epilepsy, microcephaly, macrocephaly, hypotonia), and variants (causative, putative pathogenic and contributing factors) were provided. Considering the overall clinical manifestation of our cohort, we give out the variant data and phenotypic traits of the 150 patients from CAGI5 ID-Challenge as training and validation for the prediction methods development.

## Introduction

Neurodevelopmental disorders (NDDs) are a class of disorders that affect brain development and function, characterized by significant genetic and clinical variability. Children with NDDs exhibit cognitive, behavioral, and motor developmental delays. NDDs include conditions such as autism spectrum disorder (ASD), intellectual disability (ID), attention deficit hyperactivity disorder, epilepsy, and motor disorders (Morris-Rosendahl & Crocq, 2020; Parenti et al., 2020). Multiple NDDs co-occur with brain size abnormalities, such as microcephaly and macrocephaly (Ritchie & Lizarraga, 2023). A combination of two or more of these disorders is frequently reported in patients as comorbidities, which share common functional pathways (Parenti et al., 2020).

The etiology of NDDs is associated with various genetic alterations, including chromosomal rearrangements, copy number variations, small insertions or deletions, and point mutations. Currently, the most common molecular diagnostic practice involves the use of different next-generation sequencing (NGS) approaches, such as targeted gene panels, whole exome sequencing (WES), and whole genome sequencing (WGS). Computational approaches have become crucial for the analysis of data generated by these technologies, enabling the prediction of a patient’s phenotype from their genotype and the identification of causal variants against millions of others. However, due to the genetic and clinical complexity of NDDs, a considerable number of children still lack a molecular diagnosis. Deciphering and analyzing the enormous amount of data produced WES or WGS, including genes not yet associated with the disease, is a challenge (Sun et al., 2015). Cost-effective gene panels have been widely introduced in routine clinical genetic diagnostics where the analysis of genetic data is limited to the selected genes. However, also in this case many patients will have one or more novel variants that have never been detected before. The classification of novel DNA variants is a difficult and incompletely solved problem.Nevertheless, for the progress of precision medicine, clinicians and scientists must possess the capability to draw conclusions regarding disease susceptibility and drug effectiveness based on genetic information. The interpretation of genetic data stands as a significant challenge in the practical application of precision medicine.

The Padua Genetics of Neurodevelopmental Disorders Lab at the Department of Woman and Child Health (University Hospital of Padua, Italy), collaborated with the Critical Assessment of Genome Interpretation (CAGI) (https://genomeinterpretation.org/) to create a new Intellectual Disability (ID)-challenge for the sixth edition of CAGI (CAGI6). Similar to the ID-challenge in CAGI5, which involved genetic data obtained from a panel of 74 genes applied to a cohort of 150 pediatric patients (Carraro et al., 2019), CAGI6 expanded the cohort to include 415 patients. Predictors had two primary tasks: (a) predict the phenotypes and (b) predict one or more causal variants that explain the patient’s disease phenotype. However, the challenge aimed to encourage the development of prediction methods that are as accurate as possible and can be used in future clinical practice, starting from the phenotype to identify its genetic cause or vice versa. This is especially important for complex and heterogeneous disorders.

The assessment was performed considering the clinical notes specific for each patient, collected from geneticist and candidate variants (pathogenic and potentially pathogenic) identified by the Padua NDD lab through the targeted gene-panel analysis (Aspromonte et al., 2019), submitted to the CAGI Special Issue in Human Genetics. Additionally, considering the amount of genetic data and the remaining undiagnosed cases, the assessors have taken into account the predicted variants from various groups to assess whether some overlooked variants can indeed play a key role in the patient’s phenotype (new findings).

## Methods

### Challenge format

The CAGI6 ID-challenge was divided into two tasks. First, teams were asked to predict a patient’s clinical phenotype to a broad seven phenotypic traits. Second, teams were asked to predict one or more causal variant(s) based on the data derived from the customized gene-panel sequencing of 415 pediatric patients.

The phenotypic traits for each patient are based directly on information provided by clinicians. Each patient can have one or more phenotypic traits.

Sequencing data were provided as VCF files by the Laboratory of Molecular Genetic of Neurodevelopmental Disorders, University Hospital of Padua (Italy), and were linked to a specific patient. The VCF files contain exons and flanking intron regions into 74 genes sequenced with the Ion Torrent PGM platform and processed with the Ion Torrent Suite v5.0 software, as described in (Aspromonte et al., 2019). Further information on sequence data processing is available in the VCF files (e.g., Genotype Quality, the Coverage or Called genotype). The variants have not been filtered, thus VCF files may contain sequencing errors that should be excluded by sequencing or genotype quality parameters evaluation.

The description of the seven disease phenotypes, the 74 gene identifiers, the gene captured regions in browser extensible data (BED) format, a submission template, and a submission validation script were also provided and have been submitted as in the Aspromonte et al. ((Aspromonte MC et al., 2023) in this the CAGI6 Special Issue.

### Training dataset for CAGI 6 ID-Challenge

The eight participant teams had also access before the CAGI6 challenge to a training dataset (sequencing data and patient’s clinical information from the CAGI5 ID-panel). This dataset has been published and includes phenotype and variants of 150 pediatric patients with NDDs. The predictors have access also to the workflow for the variants filtering, interpretation and classification (Aspromonte et al., 2019).

### Phenotype prediction Assessment

The evaluation of predictive ability in this assessment primarily concentrated on examining the performance of various submissions across different disease phenotypes. This problem can be referred to as a two-class prediction problem (Fawcett, 2006) and this assessment approach has been proven successful among different domains. It offers the advantage of simplifying the assessment process by enabling a comparison on the performance of different methods for each individual phenotype. This is in contrast to evaluating them based on the entire predicted class matrix (415 × 7), which consists of one prediction for each patient and phenotype.

Predicted disease classes for each submission were assessed against the clinical phenotype given in the Padua NDD lab dataset (ground truth), using the procedure described below. If the predictors failed to offer a probability value and left an asterisk on the template file, it was regarded as having a probability of zero during the evaluation.

The assessment starts, separately for each phenotype column, with the submitted probability values into binary classes conversion: positive (1) or negative (0). We determined the threshold probability value that maximized the Matthew correlation coefficient (MCC) for that particular phenotype. Subsequently, we compared all probability values for each phenotype with their corresponding threshold and assigned a value of 0 or 1 based on whether the probability was lower or higher than the threshold, respectively.

Furthermore, we employed various performance measures to evaluate the predictions for each phenotype. Sensitivity and specificity were used to assess the model’s ability to detect positive cases and discriminate between positive and negative classes. The MCC, accuracy (ACC), and F1 measures were employed to evaluate both negative and positive predictions simultaneously. Notably, the MCC has been demonstrated to be less influenced by an imbalanced dataset (Vihinen, 2012), which is the case in this challenge where some phenotypes exhibit complete imbalance.

To visualize the trade-off between the true positive rate and false positive rate, we generated receiver operating characteristic (ROC) curves by comparing the experimental and predicted probability values for each phenotype. To ensure robustness, we employed 1000 iterations of bootstrap to produce the ROC and precision-recall (PR) curves and calculate the relative AUC values (Schmidt et al., 2014). This resampling technique allows us to assess the stability and reliability of the performance estimates, especially for unbalanced datasets, and enhances the statistical validity of our results.

In order to compare the results among different predictors, we utilized the z-score at the phenotypic level on the ROC’s AUC values that we obtained with the bootstrap iterations. This statistical measure allows for the standardization of values, enabling a meaningful comparison across predictors.

### Variants prediction assessment

Predictors were also evaluated on their capacity to establish causal associations between variants and individual patients across the single nucleotide variations (SNVs) present in the provided VCF files, as part of this challenge. To evaluate this problem we can treat it as a multi-label classification problem, as for each patient we can have one or more variants selected by the Padua NDD lab dataset, and predictors were also allowed to identify zero or more variants for each patient. The Padua NDD Lab classified the selected variants in three classes, depending on their association with the disease. If a patient’s phenotype aligns with a Mendelian disorder, the variants labeled as pathogenic are seen as the direct cause behind the patient’s phenotypic manifestations, and thus classified as “disease causing (DC)”. On the other hand, variants deemed likely pathogenic (LP) or “putative” need additional examination before being classified as causative. Uncommon or new variants that are anticipated to be pathogenic and modify genes associated with a higher risk of autism have been categorized as potential contributing factors (FC), as they alone are insufficient to trigger the disease

For the assessment we will use a confusion matrix and metrics that can be extracted from it, like accuracy and recall. True positives (TP) are identified by the cases where the predictor assigned a variant to a patient that matches one of the variants associated with that patient. False positives (FP) occur when the predictor incorrectly assigns a variant to a patient, false negatives (FN), on the other hand, represent cases where the predictor fails to identify a variant that should have been associated with a patient. True negatives (TN) in this case are not evaluated, as predictors will only output variants that should be associated with the patient. Because of this, the accuracy metric is calculated with the ratio of correctly predicted variants over all available variants.

### Prediction methods

Eight teams submitted predictions, using a total of 30 models Table 1. The methods used by each team are summarized in Table 2 and described in detail below. Two of the participating teams, team 6 and team 7, participated in the ID-Challenge during the previous CAGI edition and were evaluated in the CAGI5 Assessment (Carraro et al., 2019).

### Group 1–6 models

Variant annotation, prioritization, and analysis on over 100,000 single nucleotide variants (SNVs) across 415 Variant Call Format (VCF) files was performed. We used a software called EVIDENCE (Seo et al., 2020) and followed ACMG guidelines for the classification process. Rare variants (gnomAD frequency < 5%) were annotated using Ensembl Variant Effect Predictor (VEP) and prioritized based on gene function, inheritance pattern, and relevance to disease databases (OMIM, ClinVar, and UniProt). Common SNVs (gnomAD frequency > 5%) were filtered out. For phenotype matching, patient-gene matrix scores and HPO terms were utilized. Six different models for prediction and analysis were used.

Model 1, utilized a random forest approach with a polygenic risk scoring concept, considering multiple variants contributing to a single disease. Model 2, involved a random forest model with hyperparameter tuning, modifying options to optimize the model’s performance. Model 3, focused on variants affecting canonical transcripts, training random forest models with their pathogenicity scores. Model 4, used prior probabilities and odds calculated from pathogenicity scores to calculate post probabilities. Model 5, implemented a simple scoring method based on an in-house database, assigning weight scores based on variant-phenotype relevance. Model 6, identified an optimal threshold for pathogenicity scores to improve machine learning model training.

Each model was applied to predict the 415 VCF files, and their respective approaches and findings were summarized for seven phenotypes. We explored and optimized prediction methods for variant analysis and classification, considering the influence of different factors such as polygenic risk, hyperparameter tuning, transcript selection, prior probabilities, in-house database, and threshold optimization.

### Group 2–6 models

Based on the provided BED file and the hg19 genome sequences we extracted the DNA sequences for the 74 genes. The mutated sequences for each patient were then derived from the VCF files. To obtain sequence-wise representations for the genes, we utilized DNABERT (Ji et al., 2021), followed by a k-mer tokenization and ultimately settled on a value of 6 for k. Next, we constructed a graph representation for each sample, where the genes served as nodes. The adjacency matrix was defined by a 6-channel gene-gene interaction (GGI) network, as applied in (Karimi et al., 2020). We then employed a graph convolutional network (GCN) (Kipf & Welling, 2017) for message passing and a learnable weighted summation layer for the graph-wise representations. Finally we used a Multilayer Perceptron (MLP) with a 7-dimensional sigmoid layer to predict the labels of the target diseases. The provided labeled samples were randomly divided into training and test sets using a 7:3 ratio. We tuned the models by evaluating the test performance and developed 6 versions with distinct configurations. For each version we identified the top-20 variants based on the attention weights as the candidate variants. We tried various combinations as ensemble models and determined the causal variants by voting based on their frequency among the candidates. The results of the top-6 ensemble models were submitted at last.

### Group 3 – 2 models

The Ensembl Variant Effect Predictor (VEP) tool (McLaren et al., 2016) and the precalculated REVEL scores (Ioannidis et al., 2016) were used for annotating the raw VCF files. For variants reported with multiple REVEL scores, we selected the highest score. Variants were filtered based on the following four criteria: (1) absent or with a MAF < 5% in 1000 Genomes Project data, (2) not homozygous reference alleles, (3) present in only one sample, (4) protein-altering variants. The ClinVar databases (Landrum et al., 2014), phenotype-specific Phenolyzer score (Yang et al., 2015) and REVEL score were used to prioritize variants. The variant reported as pathogenic/likely pathogenic in ClinVar or the top-ranked variant that combines the ranking of Phenolyzer score and REVEL score was identified as putative causative variants. Polygenic risk scores (PRS) were used to build the prediction model. To construct PRS for each of the 7 neurodevelopmental presentations, the previously associated variants were extracted by searching the NHGRI-EBI GWAS Catalog (https://www.ebi.ac.uk/gwas/), IEU Open GWAS platform (https://gwas.mrcieu.ac.uk/), and GWAS Atlas (atlas.ctglab.nl). ASD and epilepsy with existing GWAS summary statistics were eligible for our analysis. The Childhood IQ GWAS summary statistics was used for Intellectual disability. The allele effect sizes were also derived from the training dataset by logistic regression analysis for each phenotype. For each phenotypic trait, we train the logistic regression model with the training dataset and make predictions for the test dataset. The two submissions correspond to constructing PRS using existing GWAS summary statistics or using effect size estimates from the training dataset.

### Group 4 – 1 model

The method implemented by the Bologna Biocomputing Group consists of 3 steps: i) variant annotation, ii) selection of putative causative variants, iii) phenotype association. Variant annotation was performed with the Ensembl Variant Effect Predictor (VEP) (McLaren et al., 2016). We retained annotations from SIFT (Ng & Henikoff, 2003) and PolyPhen (Adzhubei et al., 2013) and the allele frequency reported in gnomAD. The putative effect of missense variants was predicted with SNPs&GO (Manfredi et al., 2022). After filtering out variants with allele frequency greater than 1%, we retained as putative causative variants the following ones: i) missense variants predicted as damaging by the three adopted methods (SIFT, PolyPhen, SNPs&GO); ii) Stop-gain variants, if altering more than 75% of the wild-type protein sequence; iii) Frameshift variants. Phenotypes associated with genes containing putative and causative variants were retrieved from Human Phenotype Ontology (HPO) (Köhler et al., 2019), manually curated data and PhenPath (Babbi et al., 2019). Six genes were excluded as they are associated with all seven phenotypes of interest. The gene-phenotype associations were used to predict the phenotypic effect of the putative causative variants, and we consequently assigned to each patient a score in a binary form (unaffected/affected) for each possible phenotype.

### Group 5–5 models

To predict the causal variant(s) and clinical phenotypes from the genomic data of patients, we first preprocessed the data, applied a quality control analysis on the variants generated from the sequencing data, and filtered out low-depth genotypes. Next, we annotated the variants with VEP (McLaren et al., 2016) and precalculated CADD scores (Rentzsch et al., 2019). For the prediction of clinical phenotypes, we used two different methods: Buckets and Evolutionary Scale Modeling (ESM) protein embeddings. The “buckets” or “genomic bins” representation groups variants by genomic locations to assign them to regions of a vector with a predefined size. We used a one-dimensional binary vector to represent regions, where “one” indicates the presence of a variant in that region. We trained different neural networks for each representation and for each phenotype separately using leave-one-out cross-validation.

For the causal variant prediction, we averaged the ESM protein representations corresponding to a single gene. We then used binary vectors with ClinVar classifications and CADD scores. We trained a model using the positive set of variants that were reported as (causative, putative, or contributing factors) and the rest are treated as negative instances. We used the Synthetic Minority Oversampling Technique to handle the highly imbalanced training data. We reported the top three predicted variants with the highest prediction scores.

### Group 6–6 models

Our team developed six automated scoring systems for the needs of this challenge. All models used the Evolutionary Action (EA) method (Katsonis & Lichtarge, 2014) for estimating the pathogenic effect of each variant. Models 1, 2, and 3, scored each sample regarding each trait, based on the predicted causal effects of variants and the known associations of genes with traits. We predicted causal variants effects using the gene inheritance patterns (dominant *de novo*, autosomal recessive, and X-linked male causality), their EA score, and their Minor Allele Frequency (MAF), which was estimated using gnomAD (Chen et al., 2022), the test data, and the training data (VCF_CAGI5). The known gene-trait associations were obtained from ClinVar (Landrum et al., 2018), DisGeNet (Piñero et al., 2017), Human Phenotype Ontology (HPO) (Köhler et al., 2019), GeneCards (Stelzer et al., 2016), and Pubmed data mining. The three models differed in EA thresholds and the source of known gene-trait associations.

Models 4, 5, and 6, computed polygenic association scores using genetic variants grouped by gene, Evolutionary Action, and MAF of the training set. We did not use any known gene-phenotype associations for these models. Model 5 was trained for male and female patients separately. Model 6 excluded training samples without trait associations. For all six models, the variants with the strongest contributions to the sample’s final score were predicted as causal.

### Group 7 – 2 models

Our approach integrated variant pathogenicity prediction with gene-disease association inference to assign weights to each variant found in a patient’s gene panel, indicative of their ability to cause a given phenotype. These combined prediction scores were then used as features in supervised, phenotype-specific random forest models to make patient-level predictions, as well as more directly to identify likely causal variants. This preliminary approach explored the feasibility of building generalizable, end-to-end data-driven methods for clinical genome interpretation.

First, we annotated the challenge data set using ANNOVAR (Wang et al., 2010) and grouped variants into seven categories based on these annotations: exonic (missense only), intronic, ncRNA_intronic, UTR3, ncRNA_exonic, UTR5, and splicing. Other classes of variants were excluded. For each gene in the panel, we defined seven variant-level features as the maximum pathogenicity prediction score within each of these categories. For missense variants in exonic regions, we included only rare variants (allele frequency < = 1% in gnomAD and used MutPred2 (Pejaver et al., 2020) for Submission 1 and REVEL (Ioannidis et al., 2016) for Submission 2. For variants in non-exonic regions, we used LINSIGHT (Huang et al., 2017). To infer gene-disease associations, we used literature-based scores from the DISEASES (Pletscher-Frankild et al., 2015) database. Specifically, we downloaded DISEASES Z-scores and min-max normalized them (over the full database) to construct a gene-phenotype matrix. We then multiplied the variant-level features with these gene-level features to construct the final patient-level feature matrix, containing combined scores for the seven categories of variants for all genes in the panel.

To train phenotype prediction models, we used the ID panel challenge data set from CAGI 5 as a training set and applied the same pre-processing and feature extraction steps as above. Individual random forest models were trained for each phenotype, using 80% of the samples for training, and 20% for validation. During the training process, a grid search method was applied to identify optimal parameters for each phenotype. For the primary task of the challenge, the output scores from these models were assumed to be the probability of a phenotype for a given patient. For the secondary task of predicting disease-causing variants for each phenotype, we selected only ultra-rare exonic variants (gnomAD allele frequency < = 2 × 10^−5^) and ranked them based on the combined variant- and gene-level scores to obtain the most likely causal variant.

### Group 8 – 2 models

The prediction method was a combination of ranking sample variants and phenotype gene correlations. Briefly, impactful variants were shortlisted through multiple filters. First, to each variant a gene independent score was assigned using VPR (Variant Prioritization), an in-house tool based on MAF (Minor Allele Frequency), evolutionary conservation, *in silico* deleteriousness predictions, and reported disease associations. Cut off scores for variants in each gene were derived from the percentile ranking of similarly scored variants in Clinvar data. Only those variants with scores above the 60 th percentile for the same gene in Clinvar data were retained. Additional quality filtering to retain only variants classified as ‘high’ or ‘medium’ quality was performed (Damiati et al., 2016). Next, phenotype-gene linking was done by querying MEDLINE abstracts and articles (Rao et al., 2020). Finally, for each sample, variants from the filtered subset were ranked by novelty or rarity in cohort, quality and variant score and grouped by gene. Gene scores were then computed as the average of 2 highest scoring variants (recessive model) and highest scoring variant (dominant model). The probability of a phenotype or phenotypes being linked to a sample was based on the ranked gene scores.

## Results

The CAGI6 Intellectual Disability (ID) panel challenge was designed to test: 1) the ability of computational methods to predict comorbid phenotypes from targeted gene-panel data, 2) the accuracy of computational methods in predicting causal variants from a set of real genetic data, and 3) the effectiveness of bioinformatics algorithms in improving gene panel data analysis for clinical practice. Additionally, groups may identify variants that were not selected by the Padua NDD Lab, and use them to genetically diagnose the condition of individuals who have not received a diagnosis.

### Overview of the Phenotype Prediction Assessment

**Figure 1:**
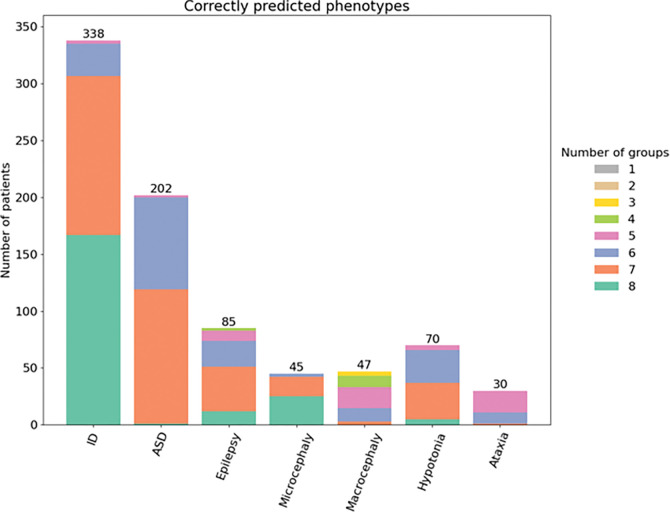
Performance of the eight groups matching the specific phenotype in 415 patients. Colors represent the proportion and number of groups which correctly predicted the phenotype.

The first part of the ID-challenge in the CAGI6, was to predict the phenotype among the seven clinical features assigned, for each patient. Figure 1 shows the number of groups that accurately predicted a patient’s phenotype when it was actually present (referred to as true positives). All groups correctly predicted the ID in 338 (49.4%) out of 352 patients; while 7 groups accurately predicted the 90.8% of patients with ID. A small number of patients (N = 45) showed microcephaly as phenotype. Eight groups correctly identified the presence of this trait in 55% of individuals and at least 7 groups correctly identified the microcephaly in 93.3% of the patients.

**Figure 2:**
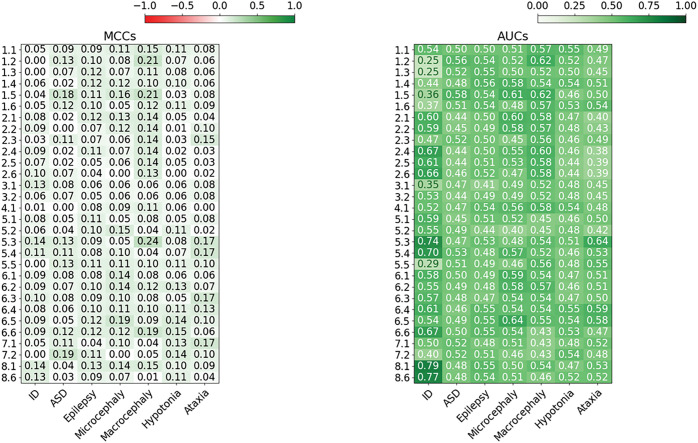
Overall performance for each submission on phenotype prediction. (A) Each cell represents the mean AUC values of the ROC for the 1000 bootstrap iterations. The color scale ranges from dark (+1, perfect performance) to white (0, bad performance). White means random performance. (B) Each cell represents MCC values. The color scale ranges from green (+1, perfect correlation) to red (−1, negative correlation). White means no better than random prediction. AUC, area under ROC curve; MCC, Matthew correlation coefficient.

**Figure 3:**
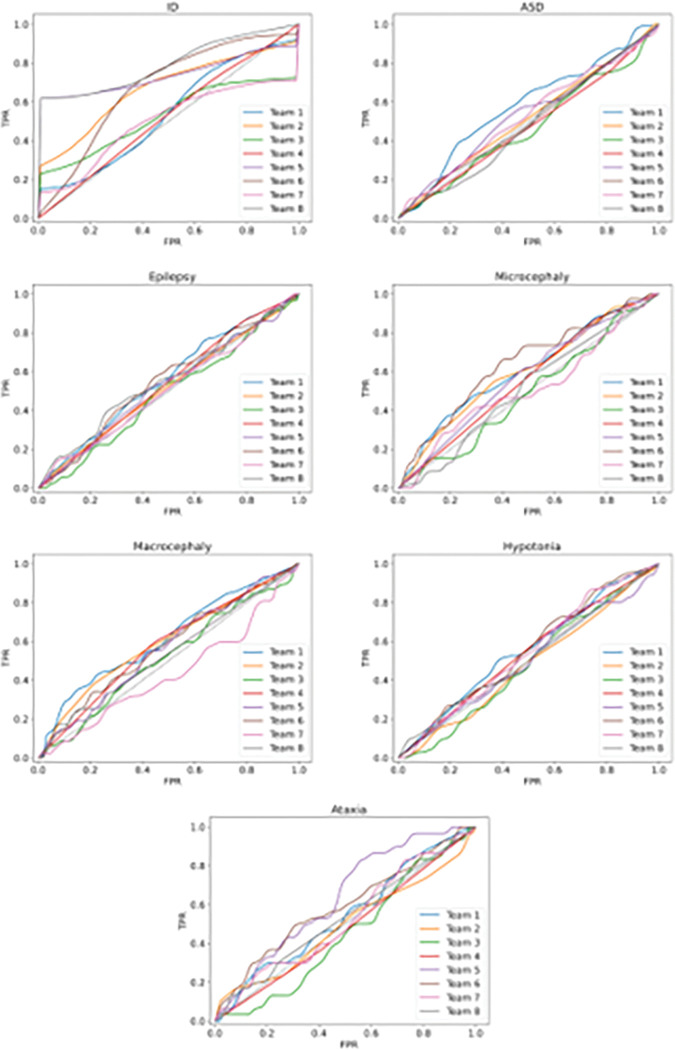
Distribution of the ROC curves for all seven clinical traits. The best performant submission for each phenotype, based on the AUC value, is shown.

The overall submission performance was assessed using the AUC for each phenotype, the maximum MCC values (Fig. 2A and B) and ROC curve (Fig. 3).

Looking at the different phenotypic traits (Fig. 2A and B), the ID phenotype was the easiest to match for SID#8.6 and SID#8.1, followed by SID#2.4 and SID#5.3. We notice that SID#8.6 accurately predicted the phenotype for 255 out of 352 patients, displaying a positive correlation with the available clinical data and achieving an F1-score of 0.85.

The second most prevalent trait in our cohort is the ASD, reported by clinicians in 202 out of 415 pediatric patients. The highest AUC values for this phenotype was attained by SID#1.5 (AUC 0.59) and SID#1.2 (AUC 0.58). Additionally, SID#5.4 ranked third with an AUC of 0.55. However, it is worth noting that the AUC values for ASD remain relatively low, approaching random performance.

When considering the MCC value to assess the phenotype predictions performance, we notice a significant difference overall compared to the AUC results, where some quite high AUC values result in lower MCC, and vice versa. The MCC is impervious to the effects of imbalanced categories and it provides a more realistic picture of prediction performance. Considering that most patients have the ID and ASD phenotypes, the confusion matrix is completely biased towards true positive values due to the highly imbalanced classes. For this reason, the ROC curve and conseguent AUC do not correctly reflect the real predictor performance, even though we applied the bootstrapping technique in order to mitigate this phenomenon.

Contrary to the previous CAGI5 (Carraro et al., 2019), we notice some differences in the phenotype predictions. In detail, all phenotypes were predicted by at least one team (Fig. 1). The prediction of the Epilepsy phenotype in CAGI6 exhibited superior performance, as evidenced by a mean MCC value of 0.09, nearly twice the previous value. Notably, SID#1 attained the highest results, achieving an AUC of 0.58, an MCC of 0.12, and an F1 score of 0.38 for SID#4 (Fig. 2A and B). These performances, when considering all submissions and groups, stand out as the most performant.

For the microcephaly and macrocephaly any improvement was not observed, but it is important to specify that the CAGI5 dataset included only 18 patients with microcephaly and 12 with macrocephaly. In contrast, considering the larger cohort in the CAGI6 dataset, more than double of patients have been reported with these specific phenotypes (Table 3). Nevertheless, certain submissions demonstrated accurate prediction of the patient phenotypes, such as the SID#6.5 for microcephaly achieving an AUC of 0.64 and a recall of 0.67, and SID#1.5 for macrocephaly achieving an AUC of 0.65 and a recall of 0.3 (Fig. 3).

We reported that 71 patients out of 415 were affected by hypotonia Table 3, while 254 out of 415 patients resulted negative to this clinical phenotype. Compared to CAGI5, we did not notice a significant improvement for this phenotype. The maximum AUC across all submissions was 0.54, achieved by SID#1.1, attaining a recall of 0.5 and an F1 score of 0.35.

The presence of the ataxia phenotype was observed in a group of 30 patients Table 3, while 285 patients did not exhibit any signs of ataxia. The SID#5.3, has an highest-performing model with a z-score of 2.48, an AUC of 0.66, and an F1 score of 0.23. It is worth noting that this result is consistent with the previous assessment. However, it should be mentioned that fewer submissions achieved an AUC score exceeding 0.65 when compared to the previous evaluation. Considering the average z-score based on the ROC’s AUC for each phenotype we reported here the overall submission ranking in each phenotype Table 4.

**Figure 4:**
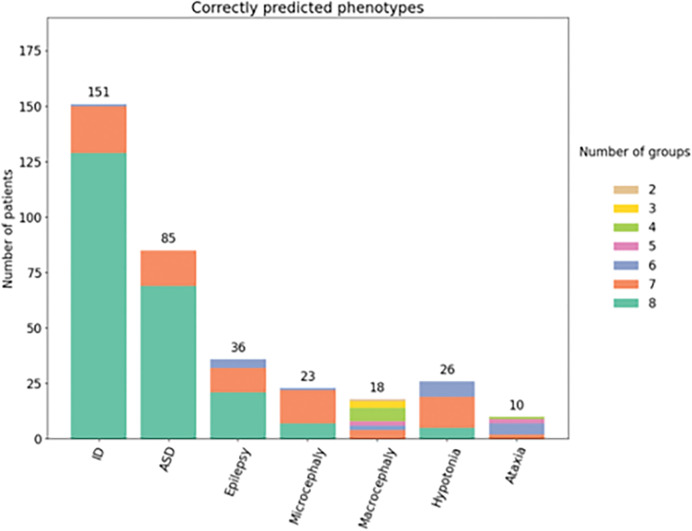
Performance of the eight groups matching the phenotype in 217 patients carrying only disease causing variants. Colors represent the proportion and number of groups which correctly predicted the phenotype in patients carrying Disease Causing

As performed in CAGI5, we also conducted an overall phenotype evaluation only for patients in whom the Padua NDD laboratory successfully identified a causative, putative variant or contributing factor. This particular subset consisted of 217 patients, accounting for 52.9% of the total cohort (Table 3). Consequently, our analysis encompassed a broader range of patients with ID, with predictions from at least one predictor in all groups accurately identifying 129 patients (85.4%). Likewise, for patients diagnosed with ASD, the predictive coverage extended to 69 patients (81.1%) (Fig. 4).

Considering this smaller subset, there were changes in the overall ranking as determined by the z-score. SID#1 achieved the top three positions, while SID#6 moved to the rank 8. Overall, the inclusion of this subset led to a 2.9% improvement in the AUC scores when considering all submissions and phenotypes (Table 5).

### Variants Prediction Assessment

**Figure 5:**
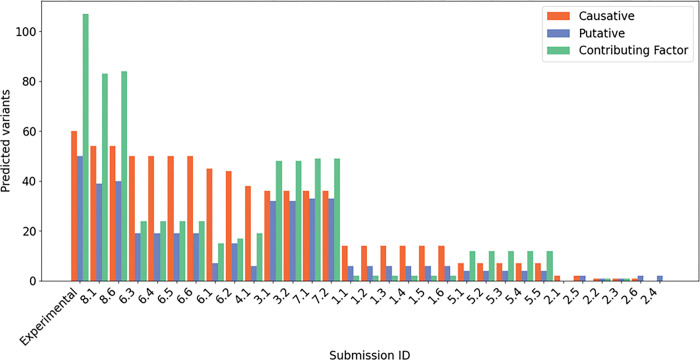
Predicted variants distribution. Category “Experimental” is the amount of variants which were identified and classified by the Padua NDD lab. Each bar represents the amount of variants and types predicted by each submission. NDD, neurodevelopmental disorder.

The second part of the CAGI6 challenge was to predict variants associated with the patient’s phenotype. Figure 5 shows the variants predicted by each submission that fall into one of the three groups considered by the laboratory, namely Causative, Putative, and Contributing Factors (CF). The first three bars of the plot show the total number of variants detected in the entire cohort with the corresponding classification.

If we consider the most clinically significant variants category, causative variants, the SID#8.1 and SID#8.6 have predicted the majority of them (54 out of 60), followed by other four groups (6, 4, 3, 7). The SID#8, SID#3 and SID#7 correctly predicted the highest number of putative and contributing factors. Variants classified as “Contributing Factors” may not perfectly adhere to the main criteria for classifying pathogenic variants, but they have been identified in genes associated with autism susceptibility. Comparing the results with the CAGI5 challenge, a major improvement can be seen, with a coverage of causative variant predictions rising from 64–90% when looking at the respective best model. Also for putative and contributing factors there has been a great improvement, from 66–79% and from 69–76%, respectively.

**Figure 6:**
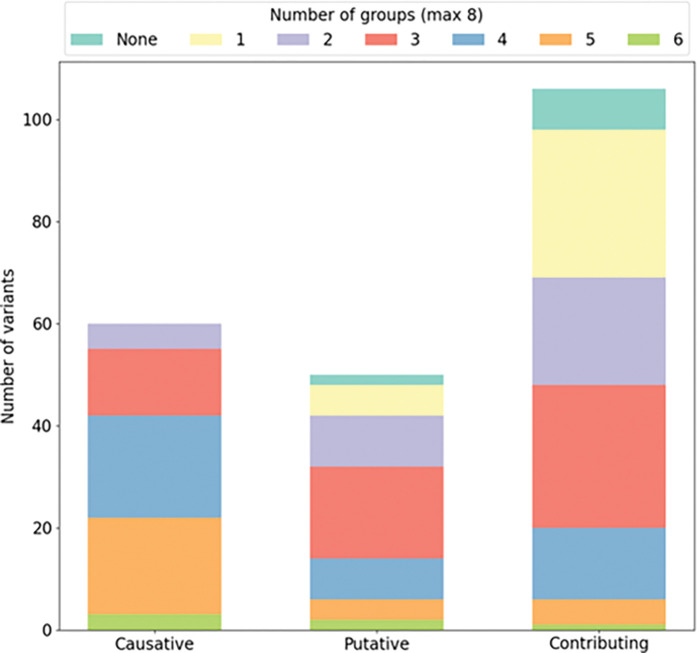
Performance of the eight groups predicting the correct variants. The amount of variants was calculated for each category (DC, LP, CF). Colors indicate the proportion and number of groups which correctly predicted those variants

In Fig. 6, we present the proportion of each mutation class predicted by different groups. We notice that total causative variants were predicted by at least two groups (violet), and a small number of variants (3 out of 60) were predicted by all groups (green). In the case of the putative variants as well, all of them have been predicted by at least one group, except for the synonyms variant p.Asn839Asn in *CNTNAP2*, which has not been prioritized by any group. However, the majority of the causative variants, have been predicted by at least 4 or 5 groups, whereas the putative variants, have been predicted by 3 or 4 groups. It can be observed that contributing factor variants are overall very sparsely predicted, where 36% of them are predicted only by one group (29 variants) or not predicted at all (8 variants). In this particular instance, no putative or contributing mutation was predicted unanimously across all groups.

Based on the data presented in Table 6, SID#8.6 emerges as the most proficient model for capturing a wide range of mutations, exhibiting a recall rate of 82%. However, its accuracy of 58% is comparatively lower, implying a significant number of false positives in the results. On the other hand, for accurate prediction, submission 6.2 surpasses all other models with an accuracy of 72.4%. Nonetheless, it exhibits a lower recall of 35%. This outcome can be attributed to the findings depicted in Fig. 5, which highlight the good performance of SID#6 in the predictions of causal variants but demonstrates its limitations in identifying putative and contributing variants.

During the assessment of the ID-challenge, it was surprising to observe that certain variants, which we defined as “difficult to predict”, were identified by multiple groups (Supplementary Table S1). Among these variants we can recognize three categories: variants with sequencing parameters indicating possible technical errors, variants that have discordant pathogenicity predictions among computational tools, and variants that affect intronic sequences far from the canonical intron-exon junctions.

As described in (Aspromonte et al., 2019), the initial step of our analysis involved filtering variants based on sequencing parameters and quality. We are reporting two variants that were confirmed as causative variants after Sanger validation and segregation analysis: p.(Arg504Gln) in *GRIN2A* and p.(Pro1585SerfsTer38) in *SHANK2*. In particular, the variant in *GRIN2A* was confirmed to be a somatic mosaicism (predicted by SID#1, 3, 7, 8), while the variant in *SHANK2* is a frameshift deletion with sequencing parameters that gave the impression the variant was an error (predicted by SID#4 and SID#8).

To prioritize rare missense variants one important step is to consider the variant’s effect through the computational methods. Our workflow includes a filtering step based on the consensus of the pathogenicity score obtained by 12 different tools and the CADD score (> 25). Three of the novel missense variants we identified in *PTCHD1*, *GATAD2B* and *ASH1L* genes, did not pass this filtering criteria. However, we have demonstrated their relevance to the disease through segregation analysis, X-inactivation and *in silico* evaluation (Aspromonte MC et al., 2023). In particular, for the case UniPD_0286, the heterozygous *GATAD2B* variant (NM_020699.4; c.922T > G; p.Cys308Gly) was predicted as causative variant by six different groups (1, 3, 4, 6, 7, 8). The deep intronic variant *MED13L* (c.4956–17A > G) was correctly predicted as a causative variant by four out of eight teams (1, 2, 5, 8). Finally, we showed with transcript analysis on the patient RNA sample that this variant creates a novel cryptic acceptor site introducing 16 intronic nucleotides in exon 22.

### Re-evaluation and classification of predicted variants

One of the objectives of CAGI6 was to detect variants that might not have been identified in the Padua NDD lab’s variant analysis but could still play a role in the patients’ phenotype. The Padua NDD lab revised more than 8000 variants. including 3016 exonic variants, 4520 intronic variants, 7 splicing variants, and 137 variants in 5’/3’-UTR (untranslated region), that were indicated by the predictors associated with at least one of the patient’s specific phenotypes. Numerous variants have been excluded mainly because they are highly prevalent both in our cohort or in the general population (gnomAD). Additionally, some of them were disregarded due to being classified as sequencing errors (Supplementary Fig. S1). Focusing on rare variants, some of them were reconsidered for Sanger validation, *in silico* or functional analysis.

In particular, for the patient UNIPD_0215, SID#1 and SID#8 teams selected the synonymous variant c.240G > A (p.Leu80Leu) in the AP1S2 gene. Patient UNIPD_0215 was referred for the gene-panel analysis at the Padua NDD lab with a suspect of Smith-Magenis syndrome. This girl presented with developmental delay, ASD, severe ID, ataxia, and some dysmorphisms (eg. synophry, large month). In addition, clinicians reported an opposite behavior and poor impulse control. Another important feature was the MRI altered with a mega cisterna magna and periventricular ischemic dilatation of the tetra ventricular system. These characteristics closely match with the syndrome associated with AP1S2, known as Pettigrew syndrome (MIM#**#** 304340). It is a severe X-linked condition that predominantly affects males. Although female carriers are generally asymptomatic. Upon further analysis with Human Splicing Finder, we found that this variant can alter splicing mechanisms, thus we re-classified it as potentially pathogenic (Aspromonte MC et al., 2023). However, further analysis such as segregation and transcript analysis are necessary to establish its pathogenic role.

## Discussion

In this study, we have reported the assessment of the new ID-challenge created for the sixth edition of the CAGI (Critical Assessment of Genome Interpretation). The NDDs Laboratory of Padua and the Biocomputing UP group from the University of Padua, have previously collaborated for the CAGI5 edition as providers and assessors of the ID-challenge. The main substantial difference between the two editions lies in the sequencing data and clinical information provided from a larger cohort (N = 415) of pediatric patients with NDDs, and the number of teams and submissions that participated in this challenge (N° teams = 8; N° submissions = 30). Two of them (SID#6 and SID#7) actively participated in the CAGI5 edition of ID-challenge as well. Before starting the CAGI6 ID-challenge, the predictors were able to access the 74-gene panel data and clinical descriptions of the CAGI5 cohort (N = 150), to train their prediction methods and find the best strategies. The predictors asked to access the sequencing data of 74 genes for the patient’s phenotype prediction and to indicate which variants infer the phenotype. The CAGI6 participants employed various methods for phenotype predictions, highlighting the diverse approaches in building disease classifiers. The majority of the methods created a gene-phenotype association matrix using known associations derived from disease databases (e.g. OMIM; HPO; GWAS) or using tools which combine several sources to associate disease terms with genes (e.g. Phenolyzer, DisGenet, PhenPath, DISEASE). Some groups curated the gene-disease list using text mining on PubMed and others built the gene-disease matrix from the CAGI5 training dataset. To build the classifier some methods explored the correlation between the prioritized variants and the gene-disease matrix, while others adopted a range of machine learning approaches such as, graph convolutional network, Random Forest, convolutional neural network. Additionally, many groups used a polygenic risk score to predict the patient phenotypes both using the GWAS data or the data derived from the training dataset. Interestingly, two of the methods that used PRS reached good performance overall or in the prediction of specific phenotype.

In variant prediction, different groups have adopted various strategies, including a standard approach that involves variant annotation and filtering based on quality and various scores of frequency. The different annotators are distinguished based on the information they use for functional annotation. For example, some approaches are based on genomic information (ANNOVAR, VEP) or using Gene Ontology (GO) terms (eg. SNP&Go). In some cases, it relies on scores derived from the combination of different tools (REVEL). Additionally, methods have been developed to determine the effect of variants by considering various software and databases for correlating the variant with the phenotype (HPO and OMIM). On the other hand, some methods take into account the inherent nature of the variants themselves, employing tools such as LoF, SIFT, PolyPhen, MutPred, and CADD Table 2.

In the phenotype prediction assessment, we applied a method similar to CAGI5 with 1000 bootstrap iterations for phenotype. Each phenotype’s probability was evaluated as 0 or 1 based on a threshold optimizing MCC. Sensitivity, specificity, and various performance measures (MCC, ACC, F1) were used. ROC curves were generated using bootstrap for stability and relative AUC calculation. The z-score at the phenotypic level allowed comparison across predictors. This approach addresses imbalanced datasets and ensures robustness and statistical validity of results.

ID and ASD were the most common phenotypes, followed by epilepsy, hypotonia, macro/microcephaly and ataxia (Table 3).

The prediction of phenotypes is complicated by the genetic and clinical heterogeneity of NDDs; the ID-challenge is different in terms of difficulty compared to a challenge based on a Mendelian inheritance disorder, such as the Hopkins challenge in CAGI4 (Chandonia et al., 2017). We have observed even in this challenge, how the phenotype prediction improves when there is a correlation between genotype and disease. We have demonstrated this in a smaller group, comprising a total of 217 patients where a causative, putative variant and/or contributing factor was identified. Therefore, the phenotype can be caused by a primary pathogenic variant, but we cannot exclude that other factors (genetic and environmental) may influence the clinical status. In some patients, the phenotype did not fully align with what expected from the alteration of the specific gene, making the diagnosis more difficult. Similar to the CAGI5 dataset, most unexpected findings were related to abnormal head size. This is the case of patients in whom causative variants have been found in the genes *WAC*, *ADNP*, *CHD8* or *MED12*, as explained in detail in (Aspromonte MC et al., 2023). There are also cases where dual genetic alterations have complicated the clinical condition. This is demonstrated by the patient with chromosome alteration, Trisomy X (UniPD_0267) and a pathogenic variant in *MECP2* (p.(Arg270Ter); or one girl that carried a pathogenic mutation in *KDM5C* (UniPD_0110) but was firstly diagnosed with fucosidosis syndrome (Leonardi et al., 2023).

Despite the difficulties in predicting phenotypes, a good result was achieved in rarer phenotypes such as microcephaly and hypotonia. The majority of patients exhibiting these clinical features were predicted by at least 6, 7, or 8 groups (Fig. 1). This means that more than half of the groups correctly predict these phenotypes.

For the variants assessment we used the confusion matrix and metrics like accuracy and recall. We assigned True Positive if the predictor matches the patient’s variant. False Positive for the incorrect variant assignment, and False Negative for missense variants predictions. Finally, the accuracy was calculated using correctly predicted variants ratio. The prediction of variants had some main protagonists. The SID#8 stood out for their predictions of variants, which we have divided into three classes (Causative, potentially pathogenic and contributing factors). Other groups also achieved good results (SID#6, SID#4, SID#3, SID#7). Some of them, considered for the filtering and variants prioritization, quality parameters or variants frequency. Many of these groups used data from CAGI5 as training for the new challenge. For example, SID#7, which achieved good predictions for both causative and putative variants, applied a method that involved excluding variants with a frequency greater than 1%. SID#3 also implemented a method by paying attention to parameters such as frequency and functional impact of variants (Fig. 5). On the contrary, SID#1, SID#2, and SID#5 seem to have some difficulties in predicting the three classes of variants. Their methods do not place much importance on variant frequency in the general population or sequencing quality to filter the hundreds of variants present in each VCF. This resulted in the selection of many variants that could be classified as sequencing errors. However, among variants predicted by these later groups, some of them were considered “difficult to predict”. Nonetheless, to infer the phenotype of the genotyped patients, some groups (SID#1, SID#3, SID#6) used a polygenic risk scoring that considered multiple variants contributing to a single disease. With this approach both rare and common variants from GWAS studies on specific phenotypic traits have been used to infer predictions.

Additionally, we noticed that SID#8, along with SID#1, indicated the variant in *AP1S2* as a causative variant. The laboratory of Padova that firstly does not consider this variant, reclassified it as potentially pathogenic on the basis of splicing prediction and phenotype consistency.

Our assessment demonstrates that there are still many limitations of the computational methods for the efficient phenotype prediction in patients affected by heterogeneous disorders like NDDs. However, the situation appears to be very different for variants. Some methods seem to be highly effective and can be improved for application in clinical practice and for the analysis of the enormous amount of data generated by WES or WGS. Furthermore, they have confirmed that the strategy of the NDDs Padua laboratory in identifying pathogenic variants has been very efficient and that both the targeted gene-panel and the data analysis workflow have yielded excellent results.

## Figures and Tables

**Table 1:** list of participating teams in CAGI6, ID-Challenge

ID	Submission ID	PI	N° models
CAGI6 ID-Challenge			
Group 1	SID#1	Anonymous	6
Group 2	SID#2	Yang Shen	6
Group 3	SID#3	Maggie Haitian Wang	2
Group 4	SID#4	Rita Casadio	1
Group 5	SID#5	Robert Hoehndorf	5
Group 6	SID#6	Olivier Lichtarge	6
Group 7	SID#7	Sean D. Mooney	2
Group 8	SID#8	Rajgopal Srinivasan	1

**Table 2:** Features of prediction methods developed by the predictors for the ID-challenge in CAGI6.

Teams				Filters					
ID	SID	CAGI5 Training	Variants Annotation	Low quality	Frequency	Variants effects	Inheritance	Gene-phenotype association	Mathematical model
1	1.1	+	EVIDENCE VEP	//	gnomAD < 5%	OMIM, ClinVar, Uniprot. EVIDENCE uses 3Cnet, spliceAI, REVEL	+	Patient-gene matrix scores, HPO	PRS
	1.2								Random forest
	1.3								Random forest
	1.4								Scoring method
	1.5								//
	1.6								//
2	2.1	+	//	//	+	DNABERT	//	//	GCN
	2.2								
	2.3								
	2.4								
	2.5								
	2.6								
3	3.1	+	VEP REVEL	//	Absent or MAF < 5% in 1000 Genomes; Hom exclusion; 1/415 pz	ClinVar, Phenolyzer, REVEL	//	GWAS	PRS
	3.2								
4	4.1	//	VEP, SNPs&GO	//	gnomAD < 1%	SIFT, PolyPhen, SNP&GO, LoF variants	//	HPO, PhenPath	Correlation
5	5.1	+	VEP, CADD	+	//	ESM, CADD	//	Buckets, ESM from training data	ML (ESM)
	5.2								
	5.3								
	5.4								
	5.5								
6	6.1	+	EA	//	MAF in gnomAD	Inheritance pattern, EA score, MAF	+	ClinVar, HPO, DisGenet, Genecard, Pubmed	Correlation
	6.2					Inheritance pattern, EA score, MAF		//	//
	6.3					Inheritance pattern, EA score, MAF		ClinVar, HPO, DisGenet, Genecard, Pubmed	Correlation
	6.4					EA, MAF		training data	PRS
	6.5					EA, MAF		//	//
	6.6					EA, MAF		training data	PRS
7	7.1	+	ANNOVAR	//	Filter in < = 1%	LINSIGHT, MutPred2, REVEL	//	DISEASES	RFC
	7.2								
8	8.1	+	In-house tool VPR	+	+	In-house tool VPR	+	ClinVar, MEDLINE	Correlation
	8.6								

Abbreviations: SID: Submission ID; VEP: Ensembl Variant Effect Predictor; CADD: Combined Annotation Dependent Depletion; MAF: Minor Allele Frequency; Hom: homozygous; LoF: Loss of Function; HPO: Human Phenotype Ontology; PRS: polygenic risk score; ESM: Evolutionary Scale Modeling; VPR: Variant Prioritization; EA: Evolutionary Action.ML: Machine Learning; GCN: Graph convolutional network; RFC: Random Forest Classifier. Mathematical models: Naive Bayes, Correlation, Bayesian, Bayesian network, Trait specific

**Table 3:** Patients for whom Padua NDD lab identified at least one causative or potentially disease variant in the answer key, summarized by phenotype.

Phenotype	Cases	Disease Causing (%)	Putative (%)	Contributing Factor (%)
**ID**	352	17	11	22
**ASD**	205	11	9	24
**Epilepsy**	84	18	7	24
**Microcephaly**	45	20	13	22
**Macrocephaly**	47	13	13	15
**Hypotonia**	71	16	11	14
**Ataxia**	30	17	13	10

*Note:* Each variant is specific for each patient and one patient can be associated with more than one phenotype. The % for each variant class was calculated by considering the total number of variants detected and classified as DC, P, or CF, out of the total number of cases exhibiting that specific phenotype trait.

*Abbreviations:* ID, intellectual disability; ASD, autism spectrum disorder

**Table 4:** Overall z-score, mean z-score and ranking among phenotypes by each submission. Highlighted in bold are the best values for each category.

Submission	ID	ASD	Epilepsy	Microcephaly	Macrocephaly	Hypotonia	Ataxia	Mean	Rank
1.1	0.02	0.42	−0.16	−0.38	0.62	**1.81**	−0.01	0.33	8
1.2	−2.00	2.09	0.98	−0.15	**1.55**	0.86	−0.37	0.42	6
1.3	−2.01	0.87	1.02	−0.42	−0.37	0.10	−0.62	−0.20	18
1.4	−0.67	−0.21	**1.49**	1.03	0.04	1.25	0.40	0.48	5
1.5	−1.23	**2.66**	0.75	1.56	1.53	−0.94	0.13	0.64	3
1.6	−1.13	0.59	0.91	−0.88	0.67	1.04	0.92	0.30	10
2.1	0.45	−1.41	−0.41	1.40	0.82	−0.51	−1.44	−0.16	17
2.2	0.38	−1.14	−0.57	1.08	0.60	−0.41	−0.92	−0.14	16
2.3	−0.48	0.77	−0.39	−1.39	0.39	−0.87	0.05	−0.27	21
2.4	0.93	−1.36	−0.25	0.36	1.13	−1.06	−1.78	−0.29	23
2.5	0.55	−1.32	−0.06	0.04	0.94	−1.60	−1.66	−0.45	26
2.6	0.83	−0.87	0.22	−0.98	0.90	−1.52	−1.69	−0.44	25
3.1	−1.26	−0.68	−2.81	−0.70	−0.36	−0.28	−0.66	−0.96	29
3.2	−0.07	−1.36	−0.70	−0.71	−0.36	−0.28	−0.68	−0.60	28
4.1	−0.08	−0.48	0.80	0.59	0.77	1.29	−0.16	0.39	7
5.1	0.37	−1.21	0.12	−0.17	−1.68	−1.05	0.22	−0.48	27
5.2	0.13	0.12	−2.06	−2.34	−1.62	−0.23	−1.21	−1.03	30
5.3	1.41	−0.44	0.50	−0.84	0.17	0.37	**2.48**	0.52	4
5.4	1.11	1.18	−0.75	0.91	−0.23	−0.84	0.66	0.29	11
5.5	−1.73	0.67	−0.54	−1.14	0.49	−0.39	0.98	−0.24	20
6.1	0.34	0.35	−0.52	1.27	0.03	−0.52	0.34	0.18	14
6.2	0.09	0.14	−0.80	1.07	0.69	−0.98	0.38	0.08	15
6.3	0.24	−0.23	−1.29	0.30	0.04	−0.76	0.13	−0.22	19
6.4	0.53	−0.82	1.07	0.24	0.05	1.77	1.72	0.65	2
**6.5**	0.07	0.13	1.14	**2.17**	0.29	1.39	1.55	**0.96**	**1**
6.6	0.97	0.22	1.08	0.24	−1.88	1.14	−0.31	0.21	13
7.1	−0.27	1.04	−0.90	−0.20	−2.00	−0.33	0.56	−0.30	24
7.2	−0.94	0.86	0.02	−1.27	−1.88	1.44	−0.21	−0.28	22
8.1	**1.79**	−0.35	1.21	−0.46	0.02	−0.64	0.70	0.32	9
8.6	1.65	−0.20	0.90	−0.24	−1.36	0.75	0.50	0.29	12

**Table 5:** Overall z-score, mean z-score and ranking among phenotypes by each submission for the smallest subset of 217 Patients. Highlighted in bold are the best values for each category.

	ID	ASD	Epilepsy	Microcephaly	Macrocephaly	Hypotonia	Ataxia	Mean	Rank
1.1	1,0554	0,4951	−0,7232	0,2439	−0,1997	2,5797	0,1666	0,5168	6
1.2	−1,447	1,2548	0,2873	1,0508	1,9385	1,8265	−0,1934	0,6739	3
1.3	−1,4175	1,065	0,3246	0,9109	−0,1034	1,2472	0,4035	0,3472	9
1.4	−0,6855	1,4784	0,1536	0,7919	−1,3593	2,1335	−0,9381	0,2249	13
1.5	−0,9367	1,7828	0,5936	1,6174	1,9256	0,2371	0,6383	0,8369	1
1.6	−0,6565	0,746	1,5397	0,4266	1,0474	0,384	1,5009	0,7126	2
2.1	0,4207	0,4161	−0,8428	−0,2424	−0,572	−0,7438	−1,9145	−0,497	24
2.2	0,4158	0,0056	−0,8408	−0,1886	−0,8788	−0,6126	−2,0077	−0,5867	26
2.3	−0,3519	−0,4046	−0,3617	−0,6245	−0,0246	−1,0238	−1,0884	−0,5542	25
2.4	1,0315	−0,3473	−0,4165	−0,8915	−0,0201	−0,7541	−1,3296	−0,3897	23
2.5	0,6566	−0,6715	−0,5579	−1,2121	−0,4267	−1,2198	−1,6471	−0,7255	27
2.6	0,9667	−1,6932	0,7497	−1,47	0,8389	−0,2145	−0,0151	−0,1196	18
3.1	−0,4284	−1,7997	−1,8717	−1,2038	0,2876	−0,9046	−0,8349	−0,9651	30
3.2	−0,7677	−0,9927	−1,5473	−1,1809	0,2999	−0,878	−0,8093	−0,8394	28
4.1	−1,0834	−0,1898	1,1253	0,881	−0,4722	1,2697	0,6918	0,3175	10
5.1	0,407	−0,4426	0,3052	−0,8148	−0,7935	−0,554	0,0852	−0,2582	21
5.2	−0,0936	−0,8955	−0,9489	−2,0982	−1,0199	−0,4099	−0,7117	−0,8825	29
5.3	0,8026	0,186	0,1246	−0,4455	1,2439	−0,5052	1,6789	0,4408	7
5.4	−0,2575	1,4701	−1,1636	1,2603	0,2976	−1,4734	−0,1552	−0,0031	17
5.5	−2,1565	−0,1089	−1,1804	−0,7565	3,0498	0,4088	1,802	0,1512	14
6.1	−0,4222	1,3225	0,2864	0,9706	−0,4952	−0,2984	0,2549	0,2312	12
6.2	−0,5273	−1,27	−0,0028	0,5717	−0,2238	−0,0368	0,5281	−0,1373	19
6.3	0,2417	0,1522	−0,2535	0,5546	0,6307	−0,8996	0,5556	0,1402	15
6.4	0,9614	0,14	−0,2506	−0,1778	−0,5748	0,6148	−0,054	0,0941	16
6.5	−0,1879	0,4222	1,2137	1,5962	−0,6986	0,2259	0,0328	0,3721	8
6.6	1,3222	−0,6826	1,5746	0,9016	−1,118	0,2046	−0,4079	0,2564	11
7.1	0,2303	0,5332	−1,3109	−0,3396	−0,5936	−1,2057	1,0226	−0,2377	20
7.2	−1,1157	0,9781	0,2302	−1,5744	−0,842	−0,3056	0,7267	−0,2718	22
8.1	2,0581	−1,824	1,8128	0,8299	−0,3802	0,3542	0,994	0,5493	5
8.6	1,9654	−1,1257	1,9514	0,613	−0,7636	0,5537	1,0251	0,6028	4

**Table 6:** Summary of variants prediction assessment by each submission. Highlighted in bold are the best recall and accuracy values.

Submission	Correctly pred. variants	Total pred. variants	Correctly pred. variants/Exp. variants (Recall)	Correctly pred. variants/Total pred. variants (Accuracy)
1.1	22	627	0.101	0.035
1.2	22	627	0.101	0.035
1.3	22	627	0.101	0.035
1.4	22	627	0.101	0.035
1.5	22	627	0.101	0.035
1.6	22	627	0.101	0.035
2.1	2	181	0.009	0.011
2.2	3	181	0.014	0.017
2.3	3	181	0.014	0.017
2.4	2	181	0.009	0.011
2.5	4	181	0.018	0.022
2.6	3	181	0.014	0.017
3.1	116	232	0.535	0.500
3.2	116	232	0.535	0.500
4.1	63	255	0.290	0.247
5.1	23	181	0.106	0.127
5.2	23	181	0.106	0.127
5.3	23	181	0.106	0.127
5.4	23	181	0.106	0.127
5.5	23	181	0.106	0.127
6.1	67	95	0.309	0.705
6.2	76	105	0.350	**0.724**
6.3	93	131	0.429	0.710
6.4	93	131	0.429	0.710
6.5	93	131	0.429	0.710
6.6	93	131	0.429	0.710
7.1	118	291	0.544	0.405
7.2	118	291	0.544	0.405
8.1	176	341	0.811	0.516
8.6	178	303	**0.820**	0.587
